# Death of M. Ollivier (D’Angers)

**Published:** 1845-06

**Authors:** 


*M. Ollivier (D'Angers.)*—M. Ollivier (D'Angers) died on the 13th of March, after a short illness, aged forty-nine. He was one of the most scientific and talented French practitioners of our times. His Treatise "On Diseases of the Spinal Cord," is universally considered to be one of the best monographs that have been written on the subject. He has also written on legal medicine, and was considered an authority on all subjects connected with that department of science. He was a member of the Academy of Medicine.—*Ibid.*

